# "A powerful intervention: general practitioners'; use of sickness certification in depression"

**DOI:** 10.1186/1471-2296-13-82

**Published:** 2012-08-09

**Authors:** Sara Macdonald, Margaret Maxwell, Philip Wilson, Michael Smith, Will Whittaker, Matt Sutton, Jill Morrison

**Affiliations:** 1 General Practice and Primary Care, Institute of Health and Wellbeing, College of Medical, Veterinary and Life Sciences, University of Glasgow, 1, Horselethill Road, Glasgow, G12 9LX, UK; 2 Mental Health, Nursing, Midwifery and AHP Research Unit, University of Stirling, Stirling, FK9 4LA, UK; 3 Institute of Health and Wellbeing, College of Medical, Veterinary and Life Sciences, University of Glasgow, Gartnavel Royal Hospital, 1055 Great Western Road, Glasgow, G12 0XH, UK; 4 Health Economics, Health Sciences Research Group, School of Community Based Medicine, University of Manchester, Jean McFarlane Building, Oxford Road, Manchester, M13 9PL, UK; 5 General Practice and Primary Care, Institute of Health and Wellbeing, College of Medical, Veterinary and Life Sciences, University of Glasgow, 1, Horselethill Road, Glasgow, G12 9LX, UK

**Keywords:** Depression, Mood disorder, Primary care, Occupational, Environmental medicine, Doctor-patient relationship, Mental health

## Abstract

**Background:**

Depression is frequently cited as the reason for sickness absence, and it is estimated that sickness certificates are issued in one third of consultations for depression. Previous research has considered GP views of sickness certification but not specifically in relation to depression.

This study aimed to explore GPs views of sickness certification in relation to depression.

**Methods:**

A purposive sample of GP practices across Scotland was selected to reflect variations in levels of incapacity claimants and antidepressant prescribing. Qualitative interviews were carried out between 2008 and 2009.

**Results:**

A total of 30 GPs were interviewed. A number of common themes emerged including the perceived importance of GP advocacy on behalf of their patients, the tensions between stakeholders involved in the sickness certification system, the need to respond flexibly to patients who present with depression and the therapeutic nature of time away from work as well as the benefits of work. GPs reported that most patients with depression returned to work after a short period of absence and that it was often difficult to predict which patients would struggle to return to work.

**Conclusions:**

GPs reported that dealing with sickness certification and depression presents distinct challenges. Sickness certificates are often viewed as powerful interventions, the effectiveness of time away from work for those with depression should be subject to robust enquiry.

## Background

Long term receipt of incapacity benefit and shorter-term sickness absence have recently been the focus of political and policy attention in the United Kingdom (UK). Sickness absence is estimated to cost £100 billion in the UK each year [1]. Across the European Union it is estimated that between 1.5 and 4% of Gross Domestic Product is lost to sickness absence [2], and in the United States between 3 – 7% of all working days are lost to sickness [3]. In many European countries the majority of sickness absence had previously been attributed to musculoskeletal disorders. A large and rapidly growing proportion of sickness absence in the UK is attributed to depression, anxiety and common mental health problems [4].

Procedures for sick-listing vary by country but in the UK, general practitioners are responsible for sickness certification and so act as gatekeepers to the work-incapacity benefits system. GPs estimate that sickness absence is raised as an issue between one and six times in each consulting session [5]. In one in every three consultations for depression, anxiety or mental ill-health, sickness certificates are issued [6]. In the area of sickness absence GPs have previously been criticised for focusing on a biomedical rather than a biopsychosocial model of health [7] and a government review of the sickness certification process concluded that it unhelpfully *“reflects an assumption that illness is incompatible with being in work.”*^*1*^*.*

Evidence that GPs hold this assumption is scarce, and rather than issuing sickness certificates ‘unthinkingly’, as some have suggested, research indicates that GPs experience quite different tensions [5,8-10]. Hussey and colleagues reported that GPs often found themselves to be unwilling intermediaries between the interests of the patient and the State, and they acknowledged that they were gatekeepers to a system they knew little about. Such tension perhaps explains why some GPs would support the removal of sickness certification from their remit^8^ and a recent review by Dame Carol Black has proposed that GPs cease to be involved in judgements around longer term sickness absence [1].

As well as the acknowledged challenges GPs face in providing sickness certification, managing common mental health problems like depression and anxiety in primary care is complex [11]. Previous research on GP decision making in relation to referrals for depression/anxiety have shown that both emotional responses and intellectual/clinical decision making processes are involved [12]. Although previous studies have considered GP perceptions of the sickness certification system, none look specifically at sickness certification in relation to depression. We, therefore, conducted a qualitative study that aimed to explore the GPs role in managing depression and work incapacity. More specifically, we asked GPs to consider their decisions regarding sickness certification and depression, how such decisions are reached, and the subsequent process to return to work or longer term incapacity. We were also interested in the potential difference between GPs in practices with high and low incapacity claimant rates.

## Methods

### Sample

The aim was to recruit a purposive sample of 30 practices from across Scotland. The rationale for using this formal purposive approach was that while GPs could speculate on the characteristics of the population they served we sought a more definitive information about their practice population. Sampling was conducted using all general practices in Scotland, based on the proportion of incapacity claimants in the practice and rates of antidepressant prescribing. Data from Scottish Neighbourhood Statistics were used to calculate incapacity levels. The level of antidepressant prescribing using defined daily doses (DDDs), was used as a proxy measure for depression, and was calculated using data provided by Information and Statistics Division Scotland.

Ethical approval for the study was granted by the West Glasgow Ethics Committee in November 2007.

### Qualitative interviews

A series of in-depth semi-structured qualitative interviews were carried out by one researcher (SM) during 2008 and 2009. GPs could opt to be interviewed by telephone if that was more convenient. A topic guide was devised to ensure that specific issues were covered in each interview but remained flexible enough to allow interviewees to introduce areas of interest to them. Questions in the topic guide reflected literature available as well as our previous work in this area [8,11]. Questions were refined by general practitioners in the research team (JM, PW) before being piloted with four general practitioners GPs. During interviews GPs were asked to discuss the decisions they make about sickness certifications when dealing with depressed patients, how short, medium and long-term absences are negotiated with patients, and the impact of depression on employment and work in the context of depressed patients’ lives.

### Analysis

Data analysis was inductive, continuous and began from the start of data collection. The analytical approach is based on the pragmatist view of grounded theory [13]. A number of a priori themes based both on the interview topic guide and previous research in the area informed the analytic process [14]. These first broad themes centred on tensions inherent in the sickness certification system, managing depression and the function of work.

Transcripts were read by two of the research team (SM and MM) and familiarisation with the data permitted additional important themes to emerge. Following discussion, a more comprehensive coding frame was developed. The coding frame was systematically applied to the data using the QSR NVivo data-handling package to catalogue and manage interview data.

We then moved to a stage of making sense of salient concepts and processes, through constant comparison of cases and to develop an understanding of any deviant cases [15].

## Results

The data confirmed previous work in this field that described the struggle that GPs experience with sickness certification, most notably the threat to their advocacy role. Emergent theory from this study is that these tensions appeared to be magnified when dealing with depressed patients and exacerbated by their difficulty in determining whether work is a help or a hindrance and the positive (and negative) effects of work as well as the positive (and negative) effects of time away from work. It was clear that GPs found it difficult to predict how individual patients might cope with work while experiencing symptoms of depression and that multiple factors, many of them non-clinical, must be considered when deciding on the most beneficial course of action for patients. Sickness certificates therefore represented a powerful intervention for GPs, and as an intervention they also carried potential side effects.

### Participants

Individual interviews were conducted with 30 (20 men and 10 women) general practitioners across Scotland [See Table 1]. Eight of the interviews were conducted on the telephone and the remainder were face-to-face, and lasted approximately one hour. Although we purposively sampled practices where there were differences in proportion of incapacity benefit claimants, we found that this did not impact on GP views or self reported behaviour *in relation to decisions around sickness certification and depression.*

**Table 1:** Summary of practice details of gps interviewed

**Number of GPs interviewed whose practices are in each cell in the sampling frame**								
High Inc.*	**High Inc.**	**High Inc.**	**Medium Inc.**	**Medium Inc.**	**Medium Inc.**	**Low Inc.**	**Low Inc.**	**Low Inc.**
Low SPR**	**Medium SPR**	**High SPR**	**Low SPR**	**Medium SPR**	**High SPR**	**Low SPR**	**Medium SPR**	**High SPR**
2	3	7	2	5	4	1	4	2
**Practice size of GPs interviewed**			**Small 1-2 partners**		**Medium 3-5 partners**		**Large 6 or more partners**	
			11		13		6	
**Average age of GPs in practices of GPs interviewed**			**≤40**	**41-45**	**46-50**	**51 – 55**	**56 – 60**	**>60**
			6	14	6	1	1	1
**% of female partners in practices of GPs interviewed**			**0**	**1-49%**	**50%**	**51-99%**	**100%**	
			7	10	6	5	2	

* High levels of incapacity in the practice from Scottish Neighbourhood statistics.

**High standardised prescribing rate for antidepressants – used as a proxy for deprivation levels.

### Advocacy and gate keeping: an inherent tension

Throughout the interviews GPs acknowledged that by virtue of their gate keeping role, everyday decisions about sickness certification have potentially far-reaching consequences that affected not only the patient but also families, employers and ultimately society. Feeling at odds with at least one, if not several, of these often competing constituencies was common. Work, though universally regarded as therapeutic in the right circumstances, could also be the source of illness and GPs had to offset the benefits with potentially harmful effects of presenteeism^a^.

"*Forcing somebody to go back to work who isn’t healthy enough is not the right thing, it’s a bad thing, in a same way taking medication that is not the appropriate medication is a bad thing (GP27)*"

The need to be mindful of the impact that symptoms of depression may have on work, to be empathic about patients’ feelings of being stigmatised, and to appreciate that patients’ difficulties may originate in the workplace was emphasised by GPs. Often these are areas where GPs feel that there is a particular need to provide additional support to their patients.

The arbitrary nature of the assessments patients undergo in order to qualify for benefits was commented on, and specifically in relation to mental health, and this reinforced the need for GPs to do what they could for patients within such a bureaucratic system

"*Where do you draw the line....., someone somewhere decided 8 points or 10 points…you get incapacity or you don’t? …or mental health …a big group of people, all of them did have a degree of mental health problems - but there was a spectrum of illness and someone arbitrarily decides you get incapacity benefit or you don’t*.(GP21)"

All GPs talked of the centrality of patient advocacy in their remit. In this context advocacy referred to being aligned with the patient as well as acting on instruction from the patient (as opposed to ‘advocacy’ in acute mental health settings where professionals are often seen as the antithesis of providing a voice for the patient). There was some variation in the extent to which GPs negotiated with patients about sickness certification but GPs felt bound by their advocacy role, which many conceded could give rise to internal conflict, as the following extracts demonstrate:

"*GPs are patients’ advocates and something comes in front of you and you have got a 50 / 50 choice whether you give a sick line or not. As an advocate they [the GP] can do as they want because you are not their employer, and I’m sure there are some times I’m doing the right thing for the patient but not the right thing for the workforce or society, or possibly the patient. But they [patients] want it [sick line] and despite discussion they’ll get it, and having that place in society where the doctor moniker, using that status to decide whether someone is fit for work or not is not always a medical decision and is sometimes quite clear, if someone breaks a leg give them an 8 week line* [certificate]*, that’s not a problem, you know ….*(GP24)"

The role of ‘gatekeeper’ within the sickness certification system is a less ambiguous task in the presence of physical ill health than it is for mental health problems. In the following extract the GP raises the tension between patient advocacy, the therapeutic nature of work and how this might threaten the GP patient relationship

"*Obviously, one, as a GP is constrained by this advocacy role that they are the patients advocate as well so that… but certainly I’ve spend many, many hours arguing with people that really the best idea for them is to continue in work or whatever rather than for them to, because it will only enhance their sense of depression if they then flunk out of a job if they are holding a job or whatever but obviously there have been people who have stomped out of here and left our surgery for good because I’ve refused to give them a line. (GP18)*"

Often patients are dealing with an array of complex and associated problems. Patients’ home lives may be worrisome; they may have caring responsibilities or have relationship difficulties with partners and/or children. It is this elaborate and individual picture that led GPs to report that patients respond to, and cope with, symptoms in different ways and the impact, therefore, of depression on work is frequently unpredictable:

"*There are so many factors in even a straight forward thing that to take even something like depression is just, it’s just impossible because some people will work through it and some people will take two weeks off or three weeks off and take anti-depressants and they will kick in and it will function fine and some people will never ever work again but you can’t, it’s really hard to pick them out. (GP 11)*"

For GPs this complexity is at odds within a sickness certification system that demands a more simple judgement.

### The therapeutic potential of time off work

Many GPs felt reluctant to describe a typical pattern of sickness absence for patients but as the following extracts demonstrate GPs were unequivocal about some presentations:

"*well there is a group of people who have got major mental health problems who are just unemployable due to that and waken up in the morning and getting through that day is enough of a challenge, it just the concept of having to go to work just isn’t an option and the majority of them, there is obviously a small percentage of them who’ve got psychosis or schizophrenia but the majority have got major personality disorders, severe anxiety, severe agoraphobia, mainly due to their upbringing where they were beaten up, abused, parents where alcoholics or whatever, they have got self esteem issues and they just haven’t got the capacity to develop normal relationships with people in the work place. There is a huge group of them who I would suggest are unemployable and they are not resistant they are just unemployable. (GP14)*"

"*People with true and straightforward depression are straightforward and work sometimes, gap time from work is sometimes worthwhile mainly because their concentration and their poor state in other things is actually making it difficult for them to function. I think if you have a straightforward depression it is usually quite obvious that a short gap and I that’s what patients feel as well but I think the big problem with depression is the complex things that people often have as associated problems you know.(GP15)*"

GPs reported that most do take some time off work and return fairly quickly. GPs were characteristically supportive of patients having a short time away from work to provide some much needed ‘breathing space’. Indeed most GPs thought it necessary to provide some short respite early on in the patients’ illness:

"*“Work is something that you can actually put into a lay-by for a fortnight or a month until you get going on medication and start to feel a wee bit more confident that you can and are able to manage. It’s quite a reasonable thing, I think time away often helps people to stay in jobs, take time off for a wee while and get them back quickly.”* (GP2)"

GPs generally thought it reasonable for patients to take some time off, and this was often attributed to the latency before antidepressant medicines became effective. Implicit in the discussions was that for the majority of patients this approach was helpful in reducing the overall burden of sickness:

"*Generally they get back to where they were, the problem is dealt with. They are on an antidepressant, they go for counselling or both and eventually go back to work. If they are off work, they are off for a couple of weeks, a month or six weeks but they go back to work, they don’t stay off. I could think of less than a handful that are off for prolonged period (GP21)*"

GPs perceived an increasing trend towards patients presenting with ‘work-related’ stress. Such difficulties ranged from bullying and harassment to simply being unable to cope with increased demands and pressure at work. Where patients’ problems stemmed from a problem at work, some GPs felt that sickness certification served an important function: they offer a catalyst for patients to discuss challenging aspects of their work with employers or superiors. In the following extract one GP describes how he explains this:

"*I say “How do you want me to write this? This can cause problems, or may cause an issue which might be good, might be bad. It might be good because it will highlight to senior management or the personnel department that your immediate boss is causing problems.” I offer it to them and say this may have implications. Some say no and some say “Yes brilliant, I want it to come to light’* (GP14)"

The type of work was important. Certain types of employment may be more prone to absence, particularly in low paid and un-skilled sectors:

"*We have a large employer here, I can’t give you the name, which is a call centre and clearly it is a very difficult place to work, it is a boring frustrating job with lots of sickness and a lot of long term sickness and, we’ve seen in the last few months … they have obviously had to address this issue at their company and they brought in some external divisional health experts who are doing things like full medicals, motivational interviewing, financial rewards, offering flexible return packages and it seems to be working very well. (GP 25)*"

Experiencing mental health problems also impacts on patient help seeking behaviour. With depression, patients may have been experiencing symptoms for some time but attempted to maintain normality, and ‘hold work together’. GPs described how patient recognition of their loss of ability to cope with work, or ‘struggling at work’, often provides the trigger for help-seeking:

"*One of the reasons is, because they are not actually coping at work and that is ….very distressing and [they say] “Well that’s the reason why I came” and maybe things have been going on at home for ages but when it’s finally affecting their work then they decide that you know they need to come …(GP16)*"

GPs also talked about patients being reluctant to take time off work because they do not want to burden colleagues’ workloads, Yet, patients may reach a tipping-point where it becomes more difficult to sustain work and fairly quickly work becomes an additional pressure. In such situations GPs rationalised that impaired cognitive function may lead to impaired performance at work, which in turn exacerbates feelings of worthlessness and guilt, both common symptoms of depression. There was therefore, a therapeutic imperative to recommending time off work.

### The therapeutic potential of work

"*Chronic depression often precludes people from getting back into gainful employment, which is unfortunate because the work environment in its own right can be one thing that is likely to stimulate people into normality*”. *(GP8)*"

GPs were certain about the advantages of work, a position reiterated in all interviews. Indeed, the structure, routine and purpose that employment gives patients was thought especially relevant for those with depression. Work could provide an escape from problems at home and generally promote self-confidence and well being:

"*I don’t think it needs a reminder because I have seen what work can do for people in both ways, good and bad. If I feel that the patient will benefit from getting an occupation and more or less getting a normal life, something regular, something to get up for in the morning, then I would be the first person to encourage that. (GP1)*"

However, as the GP above states, work can be both ‘good and bad’. What emerged from the GP interviews was that notwithstanding the benefits of work, remaining in work could be detrimental for some patients. A number of factors must be taken into consideration when judging what is best for individual patients. These include the type of job, the patient’s home situation, relationship with employers, provision for occupational health input from employers.

### Sickness certificates: a powerful intervention

Dealing with the sickness certification system and depression may pose several challenges for GPs, including the testing of their advocacy role and achieving the appropriate balance between the positive and negative impact of work on a depressed patient’s illness. What emerged from the interviews was that the sickness certificate is regarded as a powerful intervention, and one which is important in the portfolio of tools available to them. One GP reflects that this is not always sufficiently recognised by colleagues

"*I think it should be the case that a sick line is a generally well considered thoughtful bit of medical intervention and I don’t think it is at the moment. It is a very useful bit of therapy, it can be enormously helpful to people to know that their doctor is of the view that they are unable to work. It can be an enormous relief for some people and can be part of the therapy of their condition, it’s a powerful tool. It’s as powerful I think as prescribing. (GP25)*"

The symbolic importance of the sickness certificate in the doctor patient relationship for this GP is clear. Yet such a powerful intervention might also have adverse effects. GPs stressed the need for the careful thought when sanctioning time away from work because there were also potentially counter-therapeutic, and even side effects associated with sickness certificates.

"*Often a patient with depression will also have anxiety ....sometimes there is the option of prescribing a short-term benzodiazepine. I don’t mind doing that occasionally but the side-effects are dreadful. And I believe that a MED 3* [sickness certificate] *is the same as for the [drug] category, that it really is a very powerful intervention which produces a very quick turn-around and makes the patient feel better, quickly, takes the pressure off them. But then the downside is that they could, as with benzodiazepines, in the same way that they become very addicted to them very easily. So my thinking is really along those lines, that a person can become addicted to sick-lines.* (GP26)."

It is for these reasons that GPs use of sickness certification, and long term sickness certification, is a carefully considered process for individual patients, taking account of their lives, whether their ability to cope at work is compromised, the types of work they do and how this affects their well-being, and the potential risks and benefits of individual and multiple sickness certificates.

## Discussion

### Summary of main findings

Sickness certification in the realm of depression generates a distinct set of problems and concerns for GPs. The perceived tensions inherent in the system were outlined by GPs and foremost amongst them was their need to align themselves with patients, often referred to as advocacy. Most GPs saw this as their primary objective. This role takes on particular resonance for patients with depression. GPs must establish, in negotiation with the depressed patient, what role work assumes in their illness experience and how work features in the planned management of depression. For patients with severe illness, work was thought to conflict with the process of recovery. However the therapeutic benefits of work for the majority of depressed patients were emphasised, but equally, GPs saw benefits in a a short time away from work for some patients but the length of absence was key. GPs and patients therefore had to reach a balance between the remedial and the more harmful impact of work. Decisions around sickness certification and the certificates themselves represent therapeutic interventions from GPs when managing depression. No obvious differences were found in GP views in areas with high or low levels of incapacity claimants, nor were there any apparent differences between male and female GPs. There was consistent agreement about the role of the GP as advocate, the need to be flexible in response to patients, the use of a certificate as an important intervention.

Our work suggests that the sickness certificate is among the powerful “medicines” available to the general practitioner. Balint’s depictions of symbolic transactions in relation to prescribing [16] focused attention on deeper aspects of the doctor patient relationship [17]. Our finding that advocacy and the preservation of their relationship with patients are uppermost in GPs’ minds complements an extensive literature on doctor patient relationships. Chew-Graham and colleagues have found that the competing demands of the consultation can be challenging for GPs, who often must sacrifice their best judgement in the interests of maintaining the doctor patient relationship. Others have discussed the necessity of making the consultation and outcome ‘tolerable’ [18]. There is no doubt that emotional responses are also at play for GPs both in the conflict they sometimes experience and in the subsequent decisions they make [12]. Negotiations around sickness certificates can facilitate good patient/clinician engagement which is needed if depression is to be managed effectively. GPs and patients require a shared understanding and an agreement on the rationale for next steps and often a sickness certificate is a crucial intervention in this process. Indeed, sickness certificates act as a symbol of the therapeutic qualities of engagement, empathy and support. In placing such emphasis on the therapeutic nature of sickness certificates for depression, GPs may find it difficult to deny their patients a much-valued intervention.

### Comparison with existing literature

Depression is common in general practice and a common reason for work absence [4]. Although previous research has considered GPs views on sickness absence, little work has looked specifically at their perspectives on the management of the twin burdens of sickness absence and depression. Much of the existing evidence suggests that sickness certification is an area of conflict for GPs, and one that they find challenging for many reasons [8,9,19,20]. Previous research has shown that the system is largely patient led but that GPs tend to adopt either fixed or flexible approaches to sickness certification [8]. We found that most GPs adopted a flexible approach to sickness certification for depression because the illness often demands greater negotiation between the GP and the patient. Hussey and colleagues [8] illustrated that the flexible approach could be ‘stressful’, and throughout the interviews GPs describe tensions and conflict. While GPs in this study acknowledged that, though work is therapeutic and beneficial for health, the type of work is important. Butterworth et al’s [21] interrogation of Australian data found that although the mental health of unemployed respondents was poorer than that of those in work, it was better than those whose jobs were judged to have low ‘psychosocial quality’. Continuing to work must, therefore, sometimes be balanced against patient recovery particularly if the workplace is the origin of the stress. Our findings are at odds with those of Farrel et al. [7] who reported that employment advisors believed that GPs simply did not accept the therapeutic benefits of work. By contrast, GPs in this study frequently stressed the potentially undeniable therapeutic gain for depressed patients who remain in work. But crucially work was seen to be sometimes harmful. GPs interviewed felt that patients were now more likely to report problems at work or work place stress. Although there is some evidence that workplace stress has increased since the 1990s [22] the evidence for this trend is inconsistent [23]. Related to this are problems exacerbated by presenteeism [24], something which GPs in this study were aware could cause difficulties in the long term for patients and employers. As well as occupational issues, other patient factors are also important. Buist-Bowman and colleagues [25] looked specifically at depression and return to work across six European countries and concluded that around three-quarters of all patients return to work quickly and are most likely to do so if they have initiated treatment more promptly and taken the first-line antidepressant at the recommended doses. This confirms, as GPs in this study suggested, that patients with an array of complex problems are less likely to return to work.

### Strengths & limitations

Although both depression and sickness certification makes up a significant part of the GP workload, little is known about GP attitudes to the sickness certification system in relation to depression. This study sought to address this gap. GPs were asked to share their views about sickness certification and work generally, rather than focus on decisions regarding individual patients, which allowed a more candid discussion but we do not know if these self reported attitudes reflect their actual behaviour. Equally this may have resulted in a tendency to over-generalisation. The sampling frame ensured that views of GPs working in areas where there were both high and low levels of incapacity benefit claimants were obtained. However, ultimately GP’s agree (or not) to participate and it may be that those with strong views about sickness certification were more likely to volunteer. This may explain the homogeneity of views. Alternatively, it may be that GPs hold similar views and experiences in relation to sickness absence and depression regardless of the numbers of patients involved.

## Conclusions

Recent policy drives in the UK to reduce sickness-related absence and worklessness have focused on functionality and work capability. Explicit in this is the assumption that many of those absent from work or in receipt of benefit are able to perform some kind of work or meaningful activity. However, this also assumes that those that are absent from work are a homogenous group. As a recent review showed, interventions that treat all work absentees the same, irrespective of length of time away from work or the reason for absence are less likely to be successful [26]. Our study shows that GPs are committed to the therapeutic nature of work, but they are also committed to a flexible approach. Most are equally supportive of short periods away from work in the belief that this may promote recovery and ultimately reduces overall sickness absence. Sickness certification behaviour in relation to depression is seen by GPs as an important intervention that is potentially therapeutic in its own right. The utility of time away from work as a management tool requires more robust investigation and is especially pertinent following the introduction of the new Statement of Fitness for work or “Fit Note” where the emphasis is on functional ability rather than illness –related impairment [27,28].

## Endnotes

^a^Presenteeism refers to employees who come to work in spite of illness but their presence does not necessarily constitute productivity and may also be detrimental to the workplace.

## Abbreviations

GP, General Practitioner.

## Competing interests

The authors declare that they have no competing interests.

## Authors contribution

All of the authors conceived of the study, and participated in its design. SM and MM carried out the data analysis. SM, MM, PW, MS and JM helped to draft the manuscript. All authors read and approved the final manuscript.

## Pre-publication history

The pre-publication history for this paper can be accessed here:

http://www.biomedcentral.com/1471-2296/13/82/prepub
